# What Influences the Association between Previous and Future Crashes among Cyclists? A Propensity Score Analysis

**DOI:** 10.1371/journal.pone.0087633

**Published:** 2014-01-29

**Authors:** Sandar Tin Tin, Alistair Woodward, Shanthi Ameratunga

**Affiliations:** Section of Epidemiology and Biostatistics, School of Population Health, University of Auckland, Auckland, New Zealand; Arizona State University, United States of America

## Abstract

**Background:**

It is known that experience of a previous crash is related to incidence of future crashes in a cohort of New Zealand cyclists. This paper investigated if the strength of such association differed by crash involvement propensity and by the need for medical care in the previous crash.

**Methods:**

The Taupo Bicycle Study involved 2590 adult cyclists recruited in 2006 and followed over a median period of 4.6 years through linkage to four national databases. The crash involvement propensity was estimated using propensity scores based on the participants' demographic, cycling and residential characteristics. Cox regression modelling for repeated events was performed with multivariate and propensity score adjustments. Analyses were then stratified by quintiles of the propensity score.

**Results:**

A total of 801 (31.0%) participants reported having experienced at least one bicycle crash in the twelve months prior to the baseline survey. They had a higher risk of experiencing crash events during follow-up (hazard ratio (HR): 1.43; 95% CI: 1.28, 1.60) but in the stratified analysis, this association was significant only in the highest two quintiles of the propensity score where the likelihood of having experienced a crash was more than 33%. The association was stronger for previous crashes that had received medical care (HR 1.63; 95% CI: 1.41, 1.88) compared to those that had not (HR 1.30; 95% CI: 1.14, 1.49).

**Conclusions:**

Previous crash experience increased the risk of future crash involvement in high-risk cyclists and the association was stronger for previous crashes attended medically. What distinguishes the high risk group warrants closer investigation, and the findings indicate also that health service providers could play an important role in prevention of bicycle crash injuries.

## Introduction

It is often proposed that previous crash or injury increases the risk of future crashes or injuries. This phenomenon known as “accident proneness” was first observed several decades ago [1] and assumes that injuries tend to cluster within persons. Since then it has been evaluated in the general population [2] as well as in a specific subgroup (e.g., school children [3], car drivers [4] and football players [5]). A recent meta-analysis reported that the observed number of individuals with repetitive injuries is higher than would be expected by chance with a pooled odds ratio of 1.40 (95% CI: 1.34–1.46) [6].

Some authors argued that previous analyses did not account sufficiently for the spread of underlying risks between individuals or groups of individuals [7]. As emphasised in the broader phenomenon of “accident liability”[8], [9], a wide range of factors at the personal, psychosocial and environmental levels may influence the risk of previous as well as subsequent injuries [10]. Multivariate regression is often used to control for confounding but its success depends on the correct specification of the association between each covariate and the outcome.

The propensity score analysis is an alternative method that can be used to adjust for confounding in observational studies [11], [12], [13], [14]. The propensity score for an individual is the probability of receiving an exposure of interest conditional on the individual's observed covariates, and this can be estimated by building a model to predict the exposure. The estimated scores can then be integrated into analysis in at least three ways: matching, stratification and regression adjustment. This approach has some important advantages over traditional regression modelling. In particular, it is possible using this new approach to balance the distribution of covariates between exposure groups without any necessity to understand complex associations between the covariates and the outcome of interest. As a result, it is possible to control for confounding by stratification on propensity score even when the number of possible covariate combinations is very large. Using this method, effect modification may be investigated in relation to propensity to receive exposure based on the covariates.

The Taupo Bicycle Study is a prospective cohort study designed to examine factors associated with regular cycling and injury risk. Our previous (regression) analysis of the study data showed a strong association between having experienced a bicycle crash prior to baseline and involvement in police or medically attended crashes during follow-up [15]. This paper investigated whether the strength of the association differed by the cyclists' crash involvement propensity and by the need for medical care in the previous crash. The crash involvement propensity was estimated using propensity scores based on the cyclists' demographic, cycling and residential characteristics.

## Methods

### Design, setting and participants

The sampling frame comprised adult cyclists aged 16 years and over who enrolled online in the Lake Taupo Cycle Challenge, New Zealand's largest mass cycling event held each November. Participants have varying degrees of cycling experience ranging from competitive sports cyclists and experienced social riders to relative novices of all ages.

Recruitment was undertaken at the time of the 2006 event for the majority of participants, as described, in detail, elsewhere [16]. In brief, email invitations, containing a hyperlink to the study information page, were sent to 5653 contestants who provided their email addresses at registration for the event. Those who agreed to participate in the study were asked about demographic characteristics, general cycling activity in the past twelve months and habitual risk behaviours with options ranging from ‘never’ to ‘always’. They were also asked about the number of bicycle crashes they had experienced in the past twelve months as well as the number of crashes that required consulting a doctor or other health professional (e.g., physiotherapist, chiropractor). The questionnaire was completed and submitted by 2438 cyclists (43.1% response rate). Another 190 cyclists were recruited from the 2008 event by including a short description about the study in the event newsletter.

### Crash outcome data

Crash outcome data were collected through record linkage to four administrative databases, covering the period from the date of recruitment to 30 June 2011.

In New Zealand, the Accident Compensation Corporation (ACC) provides personal injury cover for all residents and temporary visitors to New Zealand no matter who is at fault. The claims database is a major source of information on relatively minor injuries with over 80% of the claims relating to primary care (e.g., GPs, emergency room treatment) only [17].

The hospital discharge data contains information about inpatients and day patients discharged after a minimum stay of three hours from all public hospitals and over 90% of private hospitals in New Zealand [18]. The mortality data contains information about all deaths registered in the country [19]. Diagnoses in each hospital visit and underlying causes of death are coded under ICD-10-AM. Bicycle crashes were identified using the E codes V10-V19. Readmissions were identified as described previously [20] and excluded.

In New Zealand, it is mandatory that any fatal or injury crash involving a collision with a motor vehicle on a public road be reported to the police. The crash analysis system data contains information on all police-reported bicycle collisions involving a motor vehicle.

For each participant, bicycle crashes identified across different databases were matched based on the date of crash allowing for a two-day difference, so as to avoid double-counting of the same crash.

### Analyses

The study sample was restricted to 2590 participants who were resident in New Zealand at recruitment. The participants were identified as having previous crash experience if they reported in the baseline survey that they had had one or more bicycle crashes in the past twelve months. Five participants with missing data on this variable were excluded.

All analyses were performed using SAS (release 9.2, SAS Institute Inc., Cary, North Carolina). Baseline data were presented as means with standard deviations and medians with interquartile ranges for continuous variables and percentages for categorical variables. All the data were complete for 2438 participants (94.3%). Missing values were computed using multiple imputation with 25 complete datasets created by the Markov chain Monte Carlo method [21], incorporating all baseline covariates and injury outcomes. Crude and adjusted differences in baseline characteristics between the participants who reported having previous crash experience and the rest of the cohort were assessed using PROC GLM. Differences were adjusted for quintiles of the propensity score. The propensity score or expected probability of having experienced a crash was computed with a multivariate logistic regression model in which previous crash experience was the dependent variable and all the baseline covariates presented in Table S1 (including age, gender, ethnicity, level of education, body mass index, the amount of cycling in general, off-road, in the dark and in a bunch, cycling to work, type of bicycle, use of helmet and conspicuity aids, distraction, neighbourhood deprivation, urban-rural status and region of residence) were the independent variables. Detailed information on the associations between the baseline covariates and crash experience at baseline and during follow-up was reported in previous publications from this study [15], [16]. The participants were then ranked by their estimated propensity score and grouped within quintiles as suggested previously [22]. The propensity score was then evaluated with: (1) a reasonable Nagelkerke's r^2^ statistic as a measure of fit, (2) a c-statistic between 0.65 and 0.85 as a measure of discriminative power, (3) similarity between the predicted and observed proportion of participants with previous crash experience within quintiles as a measure of good calibration, and (4) balanced covariates within quintiles [23], [24]. It has been demonstrated that five strata are sufficient to eliminate approximately 90% of the bias in each covariate [25].

Using bicycle crash data extracted through record linkage, incidence rates of repeated events were calculated for the participants with previous crash experience and the rest of the cohort using the person-years approach. Confidence intervals (CI) were based on the Poisson distribution. The rates were then presented by quintiles of the propensity score. The participants were censored on 30 June 2011 or date of death.

Cox proportional hazards regression modelling for repeated events was carried out using a counting process approach to assess hazards of subsequent crash involvement associated with previous crash experience. The model took into account all crash events experienced during follow-up. For the first event, the survival time was the time to the first event (or time until censored). For all later events, the survival time was the time from the previous event to the next event (or time until censored). Hazard ratios (HR) were adjusted for all the baseline covariates mentioned in Table S1, for the propensity scores and for quintiles of the propensity scores. Analyses were then stratified by quintiles and crude and propensity score adjusted HRs were calculated. In addition, previous crashes experienced were categorised by whether medical treatment was required, and similar Cox regression analyses were undertaken.

### Ethics statement

As the study used a web-based questionnaire for recruitment, informed consent was obtained electronically. All eligible participants were provided with detailed information about the study including the record linkage procedure. At the end of the information sheet, they could click a Yes/No button that says “I have read the information sheet and consent to take part in the Taupo Bicycle Study”. Clicking the “Yes” button was regarded as consent and only those who did so were taken to the next page containing the study questionnaire. This information was stored electronically for all records. Informed consent from a third party was not sought as the study sample was restricted to adult cyclists. Age of potential participants was checked at the beginning of the questionnaire by asking for their birth date. Only those aged 16 and over at the time of the survey could continue to complete the questionnaire. Ethical approval for the study including the consent procedure was obtained from the University of Auckland Human Participants' Ethics Committee. Ethical approval for linkage to the claims data was also obtained from the ACC Research Ethics Committee.

## Results

Of the 2585 participants involved in this analysis, 801 (31.0%) reported that they had experienced one or more bicycle crashes in the past twelve months prior to the baseline survey. Compared to the rest of the cohort, they were younger and more likely to be university graduates and reside in an urban area (Table S1). They spent more time cycling each week and were more likely to cycle off-road, in the dark, in a bunch and for commuting but were less likely to always use fluorescent colours and reflective materials.

**Figure 1:**
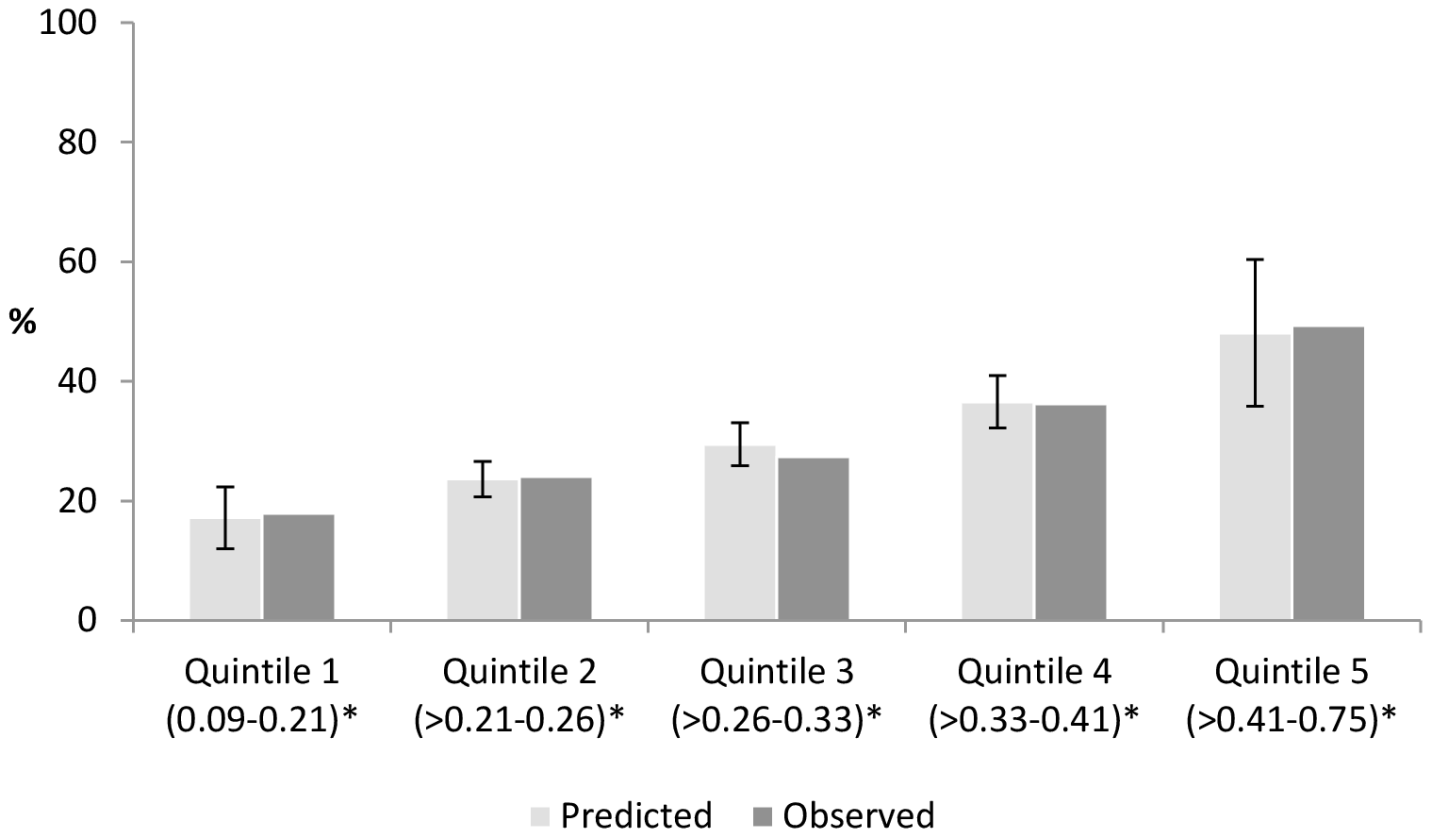
Predicted and observed probability of reporting a bicycle crash history per quintile of the propensity score. * Range of propensity scores.

The propensity score model containing age, age^2^ and all other baseline covariates had a Nagelkerke's r^2^ of 0.08 and a c-statistic of 0.65. There were 517 participants in each quintile. The mean propensity scores were 17.2%, 23.6%, 29.5%, 36.6% and 48.1% for quintile 1 to 5 respectively and were very similar to the actual proportion of participants with previous crash experience in each quintile, indicating good calibration (Figure 1). Adjustment for quintiles of the propensity score eliminated differences in baseline characteristics between participants with previous crash experience and the rest of the cohort (Table S1), suggesting that the covariates were balanced within quintiles.

During a median follow-up of 4.6 years, 324 participants with previous crash experience were involved in 520 bicycle crashes, corresponding to 146 crashes (95% CI: 133.93, 159.35) per 1000 person-years (Table 1). They had a higher risk of subsequent crash involvement even after all baseline covariates were adjusted (adjusted HR 1.25; 95% CI: 1.12, 1.40). Similar HRs were observed if propensity scores or quintiles were adjusted. There was a significant interaction between previous crash experience and quintiles (p = 0.03). When the analyses were stratified by quintiles of the propensity score, previous crash experience predicted a higher risk of future crash involvement in quintiles 4 and 5 only (Table 2).

**Table 1:** Bicycle crashes experienced during follow-up.

Number of crashes	Previous crash		No previous crash	
	N	%	N	%
1	222	27.7	355	19.9
2	53	6.6	107	6.0
3	24	3.0	41	2.3
4	12	1.5	16	0.9
5	9	1.1	5	0.3
6	3	0.4	1	0.1
7	0	0.0	1	0.1
8	0	0.0	2	0.1
9	1	0.1	0	0.0
**Total Number of crashes**	520		810	
**Rate per 1000 person-years (95% CI)**	146.23 (133.93, 159.35)		102.14 (95.22, 109.42)	
**Crude HR (95% CI)**	1.43 (1.28, 1.60)			
**Multivariable adjusted HR (95% CI)**	1.25 (1.12, 1.40)			
**Propensity score adjusted HR (95% CI)**	1.24 (1.10, 1.39)			
**Propensity quintile adjusted HR (95% CI)**	1.26 (1.12, 1.42)			

**Table 2:** Associations between previous crash experience and subsequent crash involvement per quintile of the propensity score.

Quintile of the propensity score	Rate per 1000 person-years (95% CI)		Crude HR^a^ (95% CI)	Propensity score adjusted HR^a^ (95% CI)
	Previous crash	No previous crash		
Quintile 1	85.68 (59.85, 118.88)	75.76 (63.91, 89.17)	1.13 (0.77, 1.65)	1.12 (0.77, 1.63)
Quintile 2	99.78 (75.27, 129.73)	91.77 (78.05, 107.20)	1.09 (0.78, 1.51)	1.09 (0.79, 1.51)
Quintile 3	104.15 (80.48, 132.60)	106.09 (91.08, 122.86)	0.98 (0.72, 1.33)	0.98 (0.72, 1.32)
Quintile 4	159.97 (133.96, 189.55)	109.61 (93.27, 127.99)	1.46 (1.14, 1.88)	1.45 (1.13, 1.86)
Quintile 5	204.99 (179.36, 233.25)	145.99 (124.80, 169.74)	1.40 (1.14, 1.73)	1.36 (1.10, 1.67)

a The reference group comprise participants without previous crash experience.

A total of 318 participants, that is, approximately 40.0% of those with previous crash experience, reported that they had experienced one or more bicycle crashes requiring medical care. They represented 34.0%, 33.8%, 39.6%, 40.9% and 43.8% of those with previous crash experience in quintiles 1 to 5 respectively. Table 3 shows the association between previous experience with medically versus not medically attended crashes and future crash involvement. The estimates were stronger for crashes that had received medical care (adjusted HR 1.36; 95% CI: 1.17, 1.57) than for those that had not received medical care (adjusted HR 1.20; 95% CI: 1.04, 1.37). In the stratified analyses, previous experience with medically attended crashes increased future crash risk in quintiles 3, 4 and 5, and experience with minor crashes that were not attended medically increased the crash risk in quintiles 4 and 5.

**Table 3:** Associations between previous experience with medically versus not medically attended crashes and subsequent crash involvement.

	Previous crash	
	Medically attended	Not medically attended
Crude HR^a^ (95% CI)	1.63 (1.41, 1.88)	1.30 (1.14, 1.49)
Multivariable adjusted HR^a^ (95% CI)	1.36 (1.17, 1.57)	1.20 (1.04, 1.37)
Propensity score adjusted HR^a^ (95% CI)	1.38 (1.18, 1.60)	1.14 (0.99, 1.31)
Propensity quintile adjusted HR^a^ (95% CI)	1.40 (1.21, 1.63)	1.17 (1.02, 1.34)
Crude HR^a^ (95% CI)		
Quintile 1	1.22 (0.68, 2.17)	1.08 (0.69, 1.71)
Quintile 2	1.04 (0.63, 1.73)	1.11 (0.76, 1.62)
Quintile 3	1.46 (1.00, 2.13)	0.66 (0.43, 1.02)
Quintile 4	1.49 (1.06, 2.09)	1.44 (1.08, 1.92)
Quintile 5	1.52 (1.18, 1.94)	1.32 (1.03, 1.68)
Propensity score adjusted HR^a^ (95% CI)		
Quintile 1	1.21 (0.68, 2.14)	1.04 (0.63, 1.73)
Quintile 2	1.04 (063, 1.73)	1.11 (0.76, 1.62)
Quintile 3	1.44 (0.99, 2.10)	0.66 (0.43, 1.02)
Quintile 4	1.47 (1.05, 2.07)	1.43 (1.08, 1.91)
Quintile 5	1.47 (1.14, 1.89)	1.27 (0.99, 1.61)

a The reference group comprise participants without previous crash experience.

Differences in baseline characteristics of the participants in the lowest two, mid and the highest two quintiles reflected those presented in Table S1 (Table S2).

## Discussion

### Main findings

We found that crash involvement propensity influenced the association between previous crash experience and future crash involvement in New Zealand cyclists. In the stratified analysis, the association was significant only in the highest two quintiles of the propensity score. The association was also stronger for previous crashes that had received medical care compared to those that had not.

### Strengths and limitations

In this prospective cohort study, baseline data were near-complete as mandatory fields and validation checks were incorporated in the web questionnaire. Crash outcome data were collected from four administrative databases, thereby minimising potential biases associated with loss to follow-up and self-reports.

The outcome data, however, exclude minor crashes not coming to the attention of the police or medical personnel but these events are captured in self-reported data collected at baseline. Although we were not able to estimate the association for future minor crashes, we have reported differences in the associations by the need for medical care in the previous crash. Ascertainment of crash outcomes may be affected by personal, social and health service factors [26] as well as the quality of individual data sources and record linkage [27]. Self-reported crash experience at baseline may also be affected by failure to recall [28]and socially desirable responses [29]. Likewise, self-reported covariates may not be accurate and may change over time. Nevertheless, potential misclassifications of crash outcomes and exposures tend to be non-differential in a prospective cohort study [30]. While the propensity score method was used to balance baseline covariates between exposure groups, the effect of unmeasured or unknown confounders may still be present. Finally, our participants cannot be considered representative of all New Zealand cyclists; however, this may have minimal impact on the risk estimates [31]. Importantly, the participants represented a wide variation with regard to demographics, cycling exposure and experience and the results will be valid for all cyclists and traffic environments similar to New Zealand.

### Interpretation

Our finding showing that cyclists with previous crash experience had a 43% higher risk of future crash involvement is in accordance with the existing literature [6]. However, unlike previous research, this study was able to account for (stratify over) a range of covariates including demographics, risk exposure and residential characteristics by using the propensity score approach.

In the stratified analysis, the association was significant only in quintiles 4 and 5 (where the likelihood of having experienced a crash was more than 33%), indicating that the accident (or injury) proneness phenomenon applied only to the high-risk group in this study. Cyclists in this group were younger, more likely to be males and university graduates, to cycle off-road, in the dark, in a bunch and for commuting, to engage in distracting activities such as listening to music while riding, and to reside in an urban area, and less likely to always use conspicuity aids. Explanations for “crash proneness” have included personality maladjustments, cognitive failures, stress and other mental and physical health problems [32], [33], [34], [35]. Another possibility is that crash repeaters may be more frequently exposed to environmental hazards, for example, poor road surface, poor cycling facilities and bad weather. This is beyond the scope of this paper but is worthy of further investigation.

This study found that previous experience with medically attended crashes carried a higher risk of future crash involvement than experience with other crashes, particularly in quintiles 3, 4 and 5. Crashes requiring medical care tend to be more severe, and may result in physical, psychosocial and possibly cognitive and behavioural sequelae [36], [37], [38] that affect the risk of re-injury of the same type and location [39], [40]. On the other hand, the observed association may simply reflect a higher rate of service utilisation among cyclists with previous crashes attended medically (note that crash outcome data collected during follow-up covered only those crashes that came to the attention of medical personnel or police).

Our findings suggest that Emergency Medical Services (EMS) and other health care professionals may have a role in identifying crash-prone high-risk cyclists, in delivering targeted interventions to prevent future crashes and in ensuring adequate rehabilitation before patients resume riding a bicycle. The need for EMS involvement in such activities has been recognised [41], [42], [43], [44], [45] and the success of EMS-based programs has been documented, for example, in increasing helmet use among children and adolescents [46], [47], [48]. Bicycle safety was one of the most important injury prevention activities in US emergency departments and trauma centres [49], [50]. Other studies reported that few EMS providers practise injury prevention activities [51] and some were sceptical about the appropriateness and potential impact of such activities [52]. Such information is not currently available in New Zealand.

## Conclusions

Previous experience with a bicycle crash was associated with an increased risk of subsequent crash involvement among high-risk cyclists. What distinguishes the high risk group warrants closer investigation. In addition, the association was stronger for previous crashes that had received medical care, indicating the potential role of EMS and other health service providers in prevention of bicycle crash injuries.

## Supporting Information

Table S1 **Baseline characteristics of the participants with and without previous crash experience.**
(DOCX)

Table S2 **Baseline characteristics of the participants in low, mid and high quintiles of the propensity score.**
(DOCX)
