# Supplementary material for: What Influences the Association between Previous and Future Crashes among Cyclists? A Propensity Score Analysis
Source: PLoS One. 2014 Jan 29;9(1):e87633. doi: 10.1371/journal.pone.0087633 (PMC3906177; doi:10.1371/journal.pone.0087633)
Supplement: Table S1 — Baseline characteristics of the participants with and without previous crash experience. (DOCX) [file pone.0087633.s001.docx]

## Table S1. Baseline characteristics of the participants with and without previous crash experience

| **Baseline Characteristics** |  | **Crude** | | | **Adjusted for quintiles of the propensity score** | | |
| --- | --- | --- | --- | --- | --- | --- | --- |
|  |  | **Previous crash** | **No previous crash** | **p-value** | **Previous crash** | **No previous crash** | **p-value** |
| Age | Mean (SD) | 43.0 (10.8) | 44.4 (10.2) | 0.0007 | 43.9 (10.2) | 44.0 (10.1) | 0.9 |
| Male | % | 74.3 | 71.4 | 0.1 | 72.4 | 72.3 | 0.9 |
| Maori | % | 3.3 | 4.4 | 0.2 | 3.9 | 4.1 | 0.8 |
| Education |  |  |  |  |  |  |  |
| High school (secondary) or less | % | 18.0 | 21.8 | 0.03 | 20.4 | 20.8 | 0.8 |
| Polytechnic | % | 23.9 | 26.0 | 0.3 | 25.5 | 25.4 | 0.9 |
| University | % | 57.7 | 52.1 | 0.007 | 54.1 | 53.8 | 0.9 |
| Missing | *%* | *0.5* | *0.1* |  |  |  |  |
| Body Mass Index | Mean (SD) | 25.0 (3.5) | 25.4 (3.7) | 0.003 | 25.2 (3.6) | 25.3 (3.6) | 0.7 |
| Missing | % | *0.9* | *0.4* |  |  |  |  |
| Years of cycling | Mean (SD) | 7.1 (9.0) | 6.9 (8.9) | 0.7 | 7.0 (9.1) | 7.0 (9.0) | 1.0 |
|  | Median (IQR) | 3.0 (6.0) | 3.0 (9.0) |  |  |  |  |
| Missing | % | *0.6* | *0.3* |  |  |  |  |
| Hours spent cycling per week | Mean (SD) | 6.3 (3.9) | 5.5 (3.6) | <0.0001 | 5.8 (3.5) | 5.7 (3.5) | 0.4 |
|  | Median (IQR) | 6.0 (4.0) | 5.0 (4.0) |  |  |  |  |
| Missing | % | *0.1* | *0.3* |  |  |  |  |
| % cycling off-road | Mean (SD) | 9.9 (19.4) | 7.7 (17.7) | 0.005 | 8.7 (18.2) | 8.4 (18.0) | 0.7 |
|  | Median (IQR) | 0.0 (10.0) | 0.0 (5.0) |  |  |  |  |
| Missing | % | *0.5* | *0.5* |  |  |  |  |
| % cycling in the dark | Mean (SD) | 11.2 (14.9) | 8.1 (13.4) | <0.0001 | 9.1 (12.9) | 9.0 (12.8) | 0.8 |
|  | Median (IQR) | 5.0 (19.0) | 1.0 (10.0) |  |  |  |  |
| Missing | % | *0.1* | *0.2* |  |  |  |  |
| % cycling in a bunch | Mean (SD) | 22.0 (26.0) | 18.6 (25.0) | 0.002 | 19.8 (24.8) | 19.6 (24.6) | 0.9 |
|  | Median (IQR) | 10.0 (32.0) | 5.0 (30.0) |  |  |  |  |
| Missing | % | *0.7* | *0.7* |  |  |  |  |
| Cycle to work at least once a week | % | 38.6 | 25.7 | <0.0001 | 30.9 | 30.3 | 0.7 |
| Missing | *%* | *1.9* | *2.0* |  |  |  |  |
| Type of bike most commonly used |  |  |  |  |  |  |  |
| Road | % | 86.5 | 86.7 | 0.8 | 87.0 | 86.9 | 0.9 |
| Mountain | % | 8.2 | 7.2 | 0.4 | 7.6 | 7.7 | 1.0 |
| Others | % | 5.0 | 5.6 | 0.6 | 5.4 | 5.5 | 0.9 |
| Missing | *%* | *0.3* | *0.5* |  |  |  |  |
| Always wear helmet | % | 98.6 | 98.7 | 0.6 | 99.0 | 99.0 | 0.9 |
| Missing | *%* | *0.3* | *0.4* |  |  |  |  |
| Wear fluorescent colours |  |  |  |  |  |  |  |
| Always | % | 24.0 | 31.6 | <0.0001 | 28.8 | 29.5 | 0.7 |
| Sometimes | % | 54.9 | 48.8 | 0.004 | 51.5 | 51.2 | 0.9 |
| Never | % | 20.5 | 18.8 | 0.3 | 19.7 | 19.3 | 0.8 |
| Missing | *%* | *0.6* | *0.8* |  |  |  |  |
| Ever cycle in the dark | % | 75.4 | 63.2 | <0.0001 | 67.5 | 66.8 | 0.7 |
| Always use lights | % | 82.8 | 82.7 | 0.8 | 82.6 | 82.8 | 0.9 |
| Missing | *%* | *0.0* | *0.1* |  |  |  |  |
| Use reflective materials in the dark |  |  |  |  |  |  |  |
| Always | % | 46.3 | 50.5 | 0.09 | 48.9 | 49.2 | 0.9 |
| Sometimes | % | 32.2 | 26.3 | 0.009 | 28.9 | 28.5 | 0.9 |
| Never | % | 21.2 | 22.9 | 0.4 | 22.2 | 22.3 | 1.0 |
| Missing | *%* | *0.3* | *0.4* |  |  |  |  |
| Ever listen to music while cycling | % | 17.7 | 15.7 | 0.2 | 16.7 | 16.6 | 0.9 |
| Missing | *%* | *0.5* | *0.5* |  |  |  |  |
| NZDep 2006 scores |  |  |  |  |  |  |  |
| 1-3 | % | 50.4 | 49.7 | 0.7 | 50.6 | 50.5 | 1.0 |
| 4-7 | % | 34.6 | 35.8 | 0.5 | 36.1 | 36.2 | 1.0 |
| 8-10 | % | 13.2 | 13.2 | 1 | 13.3 | 13.3 | 1.0 |
| Missing | % | 1.8 | 1.2 |  |  |  |  |
| Main urban area | % | 81.5 | 76.1 | 0.0007 | 78.7 | 78.3 | 0.8 |
| Missing | *%* | *1.8* | *1.2* |  |  |  |  |
| Region of residence |  |  |  |  |  |  |  |
| Auckland | % | 37.3 | 34.6 | 0.2 | 36.1 | 36.0 | 1.0 |
| Wellington | % | 26.0 | 18.2 | <0.0001 | 21.6 | 20.9 | 0.7 |
| Others | % | 35.0 | 45.9 | <0.0001 | 42.2 | 43.0 | 0.7 |
| Missing | *%* | *1.8* | *1.2* |  | *1.8* | *1.2* |  |

a 2006 New Zealand Deprivation Index with decile ten the most deprived neighbourhood and decile one the least.
