# Supplementary material for: What Influences the Association between Previous and Future Crashes among Cyclists? A Propensity Score Analysis
Source: PLoS One. 2014 Jan 29;9(1):e87633. doi: 10.1371/journal.pone.0087633 (PMC3906177; doi:10.1371/journal.pone.0087633)
Supplement: Table S2 — Baseline characteristics of the participants in low, mid and high quintiles of the propensity score. (DOCX) [file pone.0087633.s002.docx]

## Table S2. Baseline characteristics of the participants in low, mid and high quintiles

| **Baseline Characteristics** |  | **Quintile 1 & 2** | **Quintile 3** | **Quintile 4 & 5** | **p-value** |
| --- | --- | --- | --- | --- | --- |
| Age | Mean (SD) | 46.4 (10.3) | 44.8 (10.5) | 41.1 (10.2) | <0.0001 |
| Male | % | 65.4 | 74.6 | 78.0 | <0.0001 |
| Maori | % | 6.5 | 2.8 | 2.2 | <0.0001 |
| Education |  |  |  |  |  |
| High school (secondary) or less | % | 28.7 | 18.1 | 14.0 | <0.0001 |
| Polytechnic | % | 28.3 | 27.2 | 21.1 | 0.0004 |
| University | % | 46.6 | 54.6 | 64.9 | <0.0001 |
| Missing | *%* |  |  |  |  |
| Body Mass Index | Mean (SD) | 26.2 (3.7) | 25.2 (3.8) | 24.4 (3.7) | <0.0001 |
| Years of cycling | Mean (SD) | 6.6 (9.3) | 7.0 (9.1) | 7.4 (9.1) | 0.1 |
|  | Median (IQR) | 3.0 (7.0) | 3.0 (8.5) | 4.0 (8.0) |  |
| Hours spent cycling per week | Mean (SD) | 4.3 (3.5) | 5.5 (3.7) | 7.2 (3.6) | <0.0001 |
|  | Median (IQR) | 4.0 (4.0) | 5.0 (4.0) | 6.0 (6.0) |  |
| % cycling off-road | Mean (SD) | 4.3 (18.0) | 7.9 (18.7) | 12.9 (18.2) | <0.0001 |
|  | Median (IQR) | 0.0 (1.0) | 0.0 (5.0) | 1.0 (10.0) |  |
| % cycling in the dark | Mean (SD) | 3.2 (12.9) | 8.0 (13.1) | 15.4 (13.0) | <0.0001 |
|  | Median (IQR) | 0.0 (1.3) | 2.0 (10.0) | 10.0 (23.0) |  |
| % cycling in a bunch | Mean (SD) | 12.3 (24.8) | 19.6 (25.5) | 27.2 (25.2) | <0.0001 |
|  | Median (IQR) | 5.0 (15.0) | 10.0 (30.0) | 20.0 (47.0) |  |
| Cycle to work at least once a week | % | 8.3 | 23.5 | 56.2 | <0.0001 |
| Type of bike most commonly used |  |  |  |  |  |
| Road | % | 86.6 | 89.0 | 86.2 | 0.1 |
| Mountain | % | 6.0 | 6.9 | 9.7 | 0.004 |
| Others | % | 7.5 | 4.1 | 4.0 | 0.001 |
| Always wear Helmet | % | 99.5 | 99.0 | 98.6 | 0.1 |
| Wear fluorescent colours |  |  |  |  |  |
| Always | % | 43.7 | 28.7 | 15.3 | <0.0001 |
| Sometimes | % | 40.1 | 50.7 | 59.6 | <0.0001 |
| Never | % | 17.7 | 20.7 | 21.9 | 0.05 |
| Ever cycle in the dark | % | 40.8 | 71.9 | 90.8 | <0.0001 |
| Always use lights | % | 80.1 | 84.9 | 83.0 | 0.2 |
| Use reflective materials |  |  |  |  |  |
| Always | % | 59.5 | 53.9 | 42.5 | <0.0001 |
| Sometimes | % | 13.6 | 21.8 | 38.1 | <0.0001 |
| Never | % | 26.8 | 24.3 | 19.4 | 0.006 |
| Ever listen to music while cycling | % | 13.5 | 14.8 | 20.7 | <0.0001 |
| NZDep 2006 scores^a^ |  |  |  |  |  |
| 1-3 | % | 48.8 | 49.4 | 52.8 | 0.2 |
| 4-7 | % | 38.0 | 36.8 | 34.0 | 0.2 |
| 8-10 | % | 13.4 | 13.7 | 13.3 | 1.0 |
| Main urban area | % | 66.6 | 81.3 | 88.8 | <0.0001 |
| Region of residence |  |  |  |  |  |
| Auckland | % | 28.1 | 39.5 | 42.4 | <0.0001 |
| Wellington | % | 7.1 | 21.1 | 35.2 | <0.0001 |
| Others | % | 64.9 | 39.4 | 22.4 | <0.0001 |

a 2006 New Zealand Deprivation Index with decile ten the most deprived neighbourhood and decile one the least.
